# Bayesian spatial analysis of childhood diseases in Zimbabwe

**DOI:** 10.1186/s12889-015-2182-7

**Published:** 2015-09-02

**Authors:** Rodney Godfrey Tsiko

**Affiliations:** Department of Geoinformatics and Surveying, University of Zimbabwe, P. O Box MP167, Mount Pleasant, Harare, Zimbabwe

## Abstract

**Background:**

Many sub-Saharan countries are confronted with persistently high levels of childhood morbidity and mortality because of the impact of a range of demographic, biological and social factors or situational events that directly precipitate ill health. In particular, under-five morbidity and mortality have increased in recent decades due to childhood diarrhoea, cough and fever. Understanding the geographic distribution of such diseases and their relationships to potential risk factors can be invaluable for cost effective intervention.

**Methods:**

Bayesian semi-parametric regression models were used to quantify the spatial risk of childhood diarrhoea, fever and cough, as well as associations between childhood diseases and a range of factors, after accounting for spatial correlation between neighbouring areas. Such semi-parametric regression models allow joint analysis of non-linear effects of continuous covariates, spatially structured variation, unstructured heterogeneity, and other fixed effects on childhood diseases. Modelling and inference made use of the fully Bayesian approach via Markov Chain Monte Carlo (MCMC) simulation techniques. The analysis was based on data derived from the 1999, 2005/6 and 2010/11 Zimbabwe Demographic and Health Surveys (ZDHS).

**Results and conclusions:**

The results suggest that until recently, sex of child had little or no significant association with childhood diseases. However, a higher proportion of male than female children within a given province had a significant association with childhood cough, fever and diarrhoea. Compared to their counterparts in rural areas, children raised in an urban setting had less exposure to cough, fever and diarrhoea across all the survey years with the exception of diarrhoea in 2010. In addition, the link between sanitation, parental education, antenatal care, vaccination and childhood diseases was found to be both intuitive and counterintuitive. Results also showed marked geographical differences in the prevalence of childhood diarrhoea, fever and cough. Across all the survey years Manicaland province reported the highest cases of childhood diseases. There is also clear evidence of significant high prevalence of childhood diseases in Mashonaland than in Matabeleland provinces.

## Background

Population surveys of health and fertility provide valuable insight into the prevalence of diseases in third-world countries. Moreover, they can provide nationally and regionally representative estimates on a range of epidemiological variables, such as time of exposure, age, gender and occupation of the person exposed. The Demographic and Health Surveys (DHS) are a well-established source of reliable population data with a substantial focus on childhood diseases, which are a great deterrent militating against the demographics of under-developed nations. Zimbabwe is one such country in Sub-Saharan Africa burdened by the threat of childhood diseases. Over the past decade, Zimbabwe has been one of the least successful countries at improving child morbidity and mortality. Despite the efforts of the government to achieve goal 4 of the Millennium Development Project (MDP), which sets out to reduce the under-five mortality rate by two thirds between 1990 and 2015, one out of every eleven Zimbabwean children dies each year before their fifth birthday (approximately 35,500 children per year). Since 1990, trends in child mortality have not shown any substantial improvement, but have instead worsened when compared to other countries in different regions of the world, thus reversing the gains achieved in the previous century. The under-five mortality rate rose from 77 deaths per 1000 live births in 1994 to 84 deaths per 1000 live births as of 2011. This puts Zimbabwe within the top 50 countries in the world for high early childhood mortality. Over 65 % of these deaths occur within the first year of life, as estimated by an infant mortality rate of 57 deaths per 1000 live births. Within the first month, 31 neonates out of 1000 live births die each year. Thus, two-thirds of childhood deaths occur during infancy, with slightly more than one-third taking place during the first month of life [1].

Yet, except for some statistical descriptive reports, few systematic studies on the prevalence and treatment of common childhood diseases, which are seen as symptoms or indicators of children’s health status, causing increased mortality, have been carried out in Zimbabwe. In instances where research has been carried out, classical demographic and epidemiological studies have largely focussed on the proximate determinants associated with childhood diseases and mortality [1–6], but have made no, or limited use of the important dimension of space as an independent factor, neither have they considered the possible nonlinear effects of the risk factors governing the prevalence of childhood diseases. These limited studies use standard statistical methods such as the classical and generalized linear models that do not appropriately allow for such adjustments.

Geoadditive models, introduced by Kammann and Wand [7], will be used in this study to address this problem. These models are an alternative to the common linear models and their applications within the literature is growing [8–12]. Within the context of analysing childhood diseases; diarrhoea, cough and fever in Zimbabwe, geoadditive models account for possible non-linear effects of some factors on childhood diseases at the disaggregated provincial level that cannot be explained by the traditional set of fixed linear socioeconomic and bio-demographic factors while simultaneously controlling for geographical variation.

Such research into childhood diseases has been aided by three significant improvements. First, the increasing availability of disease and environmental data necessitates the development and application of geoadditve models to obtain valid and realistic statistical inferences that adequately describe the variation of childhood diseases. Second, spatial data of third-world countries are becoming more available, with improved coverage, quality, and variety. Third, since 1996, the DHS has consistently recorded the geographical location of each cluster of surveyed households with handheld Global Positioning System (GPS) units. This spatial information at the cluster level provides the geographic location where the affected child lives at the time of the survey and also permits linkage between DHS determinants of childhood diseases and information from other data sets. Such information has been used in some previous studies on child diseases [8, 11] and child nutritional status [13].

## Methods

### Data

The data used in this study is from the 1999, 2005–6 and 2010–11 Zimbabwe Demographic and Health Survey’s (ZDHS) undertaken by the Zimbabwe National Statistics Agency (ZIMSTAT) as part of the Zimbabwe National Household Survey Capability Programme (ZNHSCP) and the worldwide MEASURE DHS programme.

**Fig. 1:**
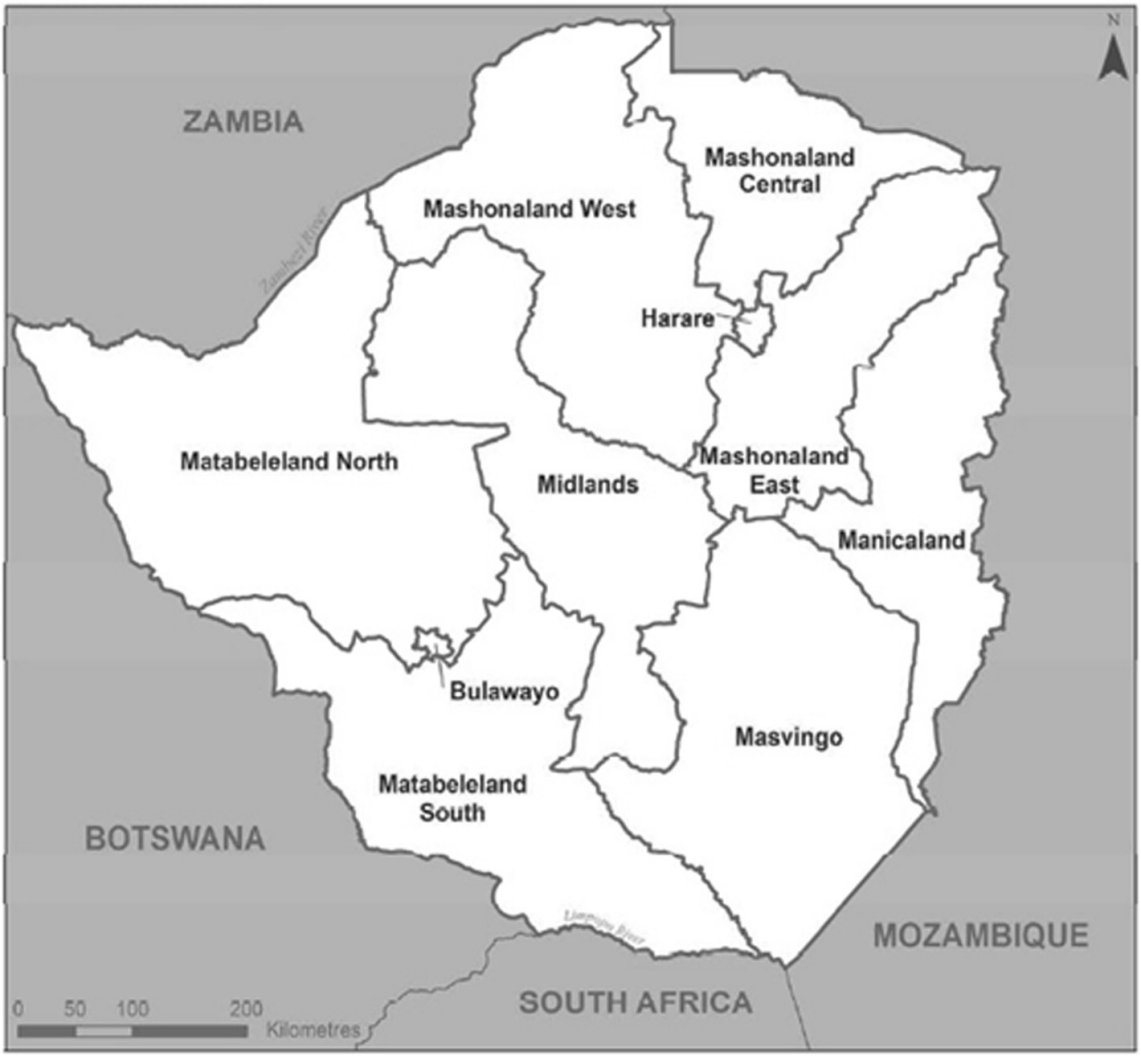
Study Area

The ZDHS samples were based on a stratified, two-stage cluster process. First, Enumeration Areas (EAs) were selected with equal probability across the 10 provinces of Zimbabwe (Fig. 1). There were 230 EAs for the 1999 survey, 400 for the 2005–6 survey, and 406 for the 2010–11 survey. Secondly, within each of these EAs, a complete household listing and mapping exercise was conducted, forming the basis of the second-stage sampling. All private households were listed. The list excluded people living in institutional households such as army barracks, hospitals, police camps etc. Households included in the survey were selected from the EA household lists, with the sample being proportional to the total number of households in the EA.

In each of the surveys, all women aged 15–49 and all men aged 15–54 who were either permanent residents of the selected households or visitors who stayed in the household the night before the survey were eligible for interview. Of interest to this study is the questionnaire used to interview all women of child-bearing age (15–49 years). It included information on working status and education of mother, sex of child, fertility patterns, socioeconomic indicators, health and care practices, health knowledge, and nutrition status of their children born within the past 5 years. The health status of children was assessed by the answer to the question: “Has your child had diarrhoea/cough/fever in the last two weeks? In less developed countries, including Zimbabwe, these three specific diseases are often symptoms for diseases such as malaria, acute respiratory infections, stomach infections, etc., that in turn are responsible for increased child mortality. Thus, it is critical to study the prevalence of childhood diarrhoea, cough or fever in the fight to combat child mortality. Table 1 shows an overview of the three diseases prior to the 1999, 2005–6 and 2010–11 surveys.

**Table 1:** Overview of childhood diseases in Zimbabwe

Year of survey	Variable	Number of observations	0:Had no disease	1:Had disease
1999	Diarrhoea	3,162	2,726	436
	Fever	3,142	2,348	794
	Cough	3,138	1,888	1,250
2005–6	Diarrhoea	4,738	4,124	614
	Fever	4,749	4,358	391
	Cough	4,713	3,675	1,038
2010–11	Diarrhoea	5,055	4,381	674
	Fever	5,048	4,533	515
	Cough	5,048	3,938	1,110

### Model specification

Given a set of observations (*y*_*i*_, *x*_*i*_, *w*_*i*_), *i* = 1, …, *n*, where *y*_*i*_ = (*diarrhoea*, *fever*, *cough*) is a vector containing separate binary responses for each disease, such that

1 $$ {y}_i=\left\{\begin{array}{cc}\hfill 1\hfill & \hfill :\kern0.22em  if\; child\; had\; diarrhoea/ fever/ cough\kern0.62em  two\; weeks\; prior\;to\; the\; survey\hfill \\ {}\hfill 0\hfill & \hfill : if\; not\hfill \end{array}\right. $$

*x*_*i*_ = (*x*_*i*1_, …, *x*_*ip*_) is a vector containing *p* continuous covariates and *w*_*i*_ = (*w*_*i*1_, …, *w*_*ir*_) is a vector of *r* categorical covariates, this study applied three separate geoadditive semiparametric Bayesian models (one for each disease) with spatial effects to estimate the probability of a child having contracted diarrhoea, cough or fever (*y*_*i*_ = 1) versus the probability of a child not having contracted any of the three diseases (*y*_*i*_ = 0). Geoadditive semiparametric Bayesian models have been described in great detail by Kamman and Wand [7], Khatab and Fahrmeir [11], and Sartorius et *al.* [14], therefore emphasis in this study will be on parts of the model pertaining only to this research.

Since the responses were binary, this study considered a logistic model to estimate the probability of a child having contracted diarrhoea, cough or fever (*y*_*i*_ = 1) versus the probability of a child not having contracted any of the three diseases (*y*_*i*_ = 0). The responses were distributed as a Bernoulli random variable such that: $f\left({y}_i\Big|{\eta}_i\right)={p}_i^{y_i}{\left(1-{p}_i\right)}^{1-{y}_i}= \exp \left[{y}_i{\eta}_i- \log \left(1+ \exp \left({\eta}_i\right)\right)\right]\kern1em for\kern0.5em {y}_i=0,1$ where *p*_*i*_ = *P*(*y*_*i*_ = 1), and *η*_*i*_ = logit(*p*_*i*_) is a parameter linked to the conventional strict linear predictor defined as follows:

2 $$ {\eta}_i={x}_i\beta +{w}_i\gamma $$

Here, *β* is a *p*-dimensional vector of unknown regression coefficients for the continuous covariates *x*_*i*_, and *γ* is a *r*-dimensional vector of unknown regression coefficients for the categorical covariates. The categorical covariates used in this study include sex of child, preceding birth interval, place of residence (urban/rural), marital status, household size, antenatal visits, place of delivery, mother’s level of education, source of drinking water, type of toilet facilities, partner’s level of education, exposure to mass media, and ever had vaccination. In addition, based on the literature (e.g., [4], [8] and [11]), the study also investigated the potentially non-linear pattern of effects of two continuous covariates, mother’s age at birth of first child (MABFC) as well as the child’s current age (CCA).

Furthermore, since the observations on childhood diseases are associated with location of residence, it was of paramount importance to account for geographical/spatial differences in the analysis. Using a convolution prior, areal level effects were introduced to allow expected spatial correlation and any unstructured areal heterogeneity of childhood diseases at the provincial level. Additional flexibility was also assumed in the predictor shown by Eq. (1) to account for possible nonlinear effects of continuous covariates. Therefore, by taking spatial and nonlinear effects into account, the strictly linear predictor shown by Eq. (1) was replaced by a more flexible semi-parametric predictor, represented by the following geoadditive regression model:

3 $$ {\eta}_i=f(MABFC)+f(CCA)+{f}_{spat}(province)+{w}_i\gamma $$

Where, *f*(*MABFC*) *and f*(*CCA*) are nonlinear smooth effects of mother’s age at birth of first child (MABFC) as well as the child’s current age (CCA), and *f*_*spat*_(*province*) is a spatial covariate function which gives information about the location of a particular observation within a province in Zimbabwe. Within the literature it is widely agreed that the spatial effect is usually a surrogate of unobserved influential factors, some of which may have a strong spatial structure and others may be present only locally (unstructured). Thus, in a further step to distinguish between the two kinds of spatial effect, *f*_*spat*_(*province*) was split into a spatially correlated (structured) part *f*_*str*_(*province*) and spatially uncorrelated (unstructured) part *f*_*unstr*_(*province*), i.e.

4 $$ {f}_{spat}(province)={f}_{str}(province)+{f}_{unstr}(province) $$

According to Besag et *al.* [15], this allows us to assess to some extent the amount of spatial dependency in the data by observing which one of the two effects is larger. If the unstructured effect exceeds the structured effect, the spatial dependency is smaller and vice versa.

Taking Eq. (4) into account, the final geoadditive model (GAM) can be expressed as:

5 $$ {\eta}_i=f(MABFC)+f(CCA)+{f}_{str}(province)+{f}_{unstr}(province)+{w}_i\gamma $$

For the above geoadditive model P-spline priors were assigned to the functions *f*(*MABFC*) *and f*(*CCA*) and for *f*_*str*_(*province*) and *f*_*unstr*_(*province*) the study utilised Markov random field prior parameters. For further details on P-spline and Markov random priors see Khatab and Fahrmeir [11], Osei *et al.* [12], Biller [16] and Sartorius et *al.* [14].

In this study, the number of districts/provinces is very limited, that is data is only available for 10 districts/provinces in Zimbabwe. As a consequence, it is expected that it would be rather difficult to separate the structured and unstructured spatial components as shown in [17] and [18], and the effects are more pronounced if the number of regions is small. As such, the models derived from Eq. (5) and used in the later stages of this study will contain either structured or unstructured spatial effects but not both in the same model.

### Model implementation

For each dataset (1999, 2005–6 and 2010–11), four separate Bayesian geoadditive models all derived from Eq. (5), were fitted to each childhood disease (diarrhoea, fever and cough). The four models were of the form shown by Eq. (6) – Eq. (9).

6 $$ M0:\eta ={\beta}_0+{z}_i^{\hbox{'}}{\gamma}_j $$

7 $$ M1:\eta ={\beta}_0+{f}_{str}(province) $$

8 $$ M2:\eta ={\beta}_0+f(CCA)+f(MABFC)+{f}_{unstr}(province)+{u}_i^{\hbox{'}}{\gamma}_j $$

9 $$ M3:\eta ={\beta}_0+f(CCA)+f(MABFC)+{f}_{str}(province)+{w}_i^{\hbox{'}}{\gamma}_j $$

In a first explanatory attempt, the data sets were fitted to a strictly linear regression model (M0) that assumed a linear effect of the bio-demographic and socio-economic categorical covariates in vector *z*, which include sex of child, preceding birth interval, place of residence (urban/rural), marital status, household size, antenatal visits, place of delivery, mother’s level of education, source of drinking water, type of toilet facilities, partner’s level of education, exposure to mass media, and ever had vaccination. Next, spatial covariates with structured effects only, were modelled at the provincial level (M1). Model M2 assumed unstructured spatial effects and nonlinear functions for the continuous covariates (child’s age and mother’s age at birth of first child) and linear effects of the categorical covariates in vector *u*, created from vector *z* by omitting factors of education (for both parents), type of toilet, exposure to mass media and source of drinking water. Model M3 is a geoadditive model, which is an extension of models M0, M1 and M2. Vector *w* in M3 includes all categorical variables in vector *z*.

The models were implemented in BayesX version 2.1 [19], an open source software for computing complex Bayesian techniques. For each model, 40,000 Markov Chain Monte Carlo (MCMC) iterations were carried out after a burn in sample of 10,000. Since in general, these random numbers are correlated, only every 20th sampled parameter of the Markov chain was stored, yielding 2000 samples for parameter estimation. Convergence was monitored by plotting trace and autocorrelation plots of the samples. Quantiles, median, mean and standard deviation for all parameters, estimated from the posterior distributions, were used to assess model fit.

Model comparison was done using Spiegelhalter et *al.* [20] classical approach, which involves a trade-off between how well a model fits the data and the level of complexity. This approach commonly known as the Deviance Information Criterion (DIC) is given by $DIC=\overline{D}+{p}_D$, where $\overline{D}$ is the posterior mean of the deviance, a measure of goodness of fit, and *p*_*D*_ is the effective number of parameters, which is a measure of model complexity and penalises overfitting. It follows that the model with the smallest DIC is estimated to be the model that would best predict a replicate dataset which has the same structure as that currently observed. According to Spiegelhalter et *al.* [21], models with differences in DIC of < 3 compared with the best model cannot be distinguished, while those between 3 and 7 can be weakly differentiated.

## Results

### Model selection

Table 2 displays the DIC values used to compare the goodness of fit of models M0, M1, M2 and M3 in explaining variations of childhood diseases, diarrhoea, fever and cough, in Zimbabwe using data from three surveys. The extension of the basic model (M0) to include non-linear effects and exclude some linear covariates in M2 significantly improved the model across all datasets. In contrast, DIC values for M1 were very high when compared to other models, signifying that for this study, structured spatial effects alone do not give a true reflection of the variation in the prevalence of childhood diseases and that other factors should be taken into account. Consequently, extension of M0, M1 and M2 to a fully additive model (M3) by including structured spatial effects, linear and non-linear effects improved the goodness of fit of the final model.

**Table 2:** Comparison of model fit using Deviance Information Criterion (DIC)

Model	1999 Survey			2005–6 Survey			2010–11 Survey		
	Deviance	P_D_	DIC	Deviance	P_D_	DIC	Deviance	P_D_	DIC
Diarrhoea									
M0	426.98	15.63	489.33	1045.88	15.75	1077.38	190.87	16.22	223.31
M1	3151.94	10.59	3173.11	4860.33	10.24	4880.81	5190.90	10.43	5211.76
M2	414.54	21.77	476.02	931.95	23.06	978.08	90.54	25.64	141.82
^a^ M3	416.02	29.24	474.50	906.67	32.15	970.97	100.45	18.94	138.32
Fever									
M0	422.65	16.23	485.88	1040.97	15.96	1072.89	190.49	16.10	222.69
M1	3130.98	11.45	3153.88	4843.40	10.67	4864.73	5185.81	10.90	5207.62
M2	417.31	28.64	474.58	926.03	21.47	968.97	90.16	26.15	142.45
^a^ M3	419.98	20.75	473.93	902.68	32.22	967.11	101.76	18.39	138.54
Cough									
M0	423.49	15.97	485.79	1036.25	16.02	1068.29	190.44	15.87	222.18
M1	3125.55	10.87	3147.29	4817.29	11.19	4839.66	5190.46	11.42	5213.30
M2	419.77	27.78	475.32	921.01	21.48	963.97	89.14	26.70	142.54
^a^ M3	420.06	19.86	473.75	896.80	32.81	962.42	99.18	19.15	137.47

^a^ *Best performing models*

Although M3 is an improvement on M2, this improvement is considered indistinguishable for differences in DIC of < 3, for example, the 1999 diarrhoea, fever and cough datasets (∆DIC values of 1.52, 0.65 and 1.57 respectively); 2005–6 fever and cough datasets (∆DIC values of 1.86 and 1.55, respectively). In such cases either model M2 or M3 can be used to best explain the prevalence of childhood diseases, however, for this study the model with the lowest DIC value was preferred. Consequently, subsequent analysis and discussions will be based on the results of M3.

### Prevalence of childhood diarrhoea

Table 3 gives the posterior means and the corresponding 95 % credible intervals for categorical covariates used to explain the variation in the prevalence of diarrhoea in children under five years of age. The effects of categorical covariates were assumed fixed, and were estimated jointly with the continuous and spatial covariates. The analysis suggests that across all survey years, making antenatal visits, giving birth in a hospital environment, and having access to a protected water source, were all associated with a reduced number of diarrhoeal incidences. There is also a suggestion that prior to the 1999 and 2005/6 surveys, male infants had a lower association with diarrhoea when compared to their female counterparts. This is in contrast to the year 2010/11 when the prevalence of diarrhoea was higher in male than female infants. Place of residence had varying degrees of association with diarrhoea between 1999 and 2010/11. Rural children were more likely than urban children to have diarrhoea in 1999, and the opposite was true for 2010/11. However, the results show no significant association between place of residence and childhood diarrhoea in 2005/6.

**Table 3:** Posterior Mean Estimates of the Fixed Effect Parameters for Diarrhoea

Covariate	Category	1999 Survey				2005–6 Survey				2010–11 Survey			
		Mean	SD	2.5 % Quantile	97.5 % Quantile	Mean	SD	2.5 % Quantile	97.5 % Quantile	Mean	SD	2.5 % Quantile	97.5 % Quantile
Const		0.125	0.059	0.011	0.239	0.161	0.047	0.067	0.257	0.440	0.315	−0.183	1.053
Sex of Child													
	Female												
	Male	−0.010	0.016	−0.041	0.022	−0.004	0.010	−0.025	0.016	0.043	0.045	−0.044	0.131
Preceding Birth Interval													
	25 + months												
	<25 months	−0.050	0.024	−0.096	−0.002	−0.010	0.015	−0.038	0.018	0.005	0.058	−0.110	0.118
Place of Residence													
	Rural												
	Urban	−0.021	0.024	−0.066	0.028	−0.006	0.019	−0.042	0.032	0.031	0.054	−0.079	0.133
Marital Status													
	Married												
	Single	−0.011	0.028	−0.065	0.043	0.033	0.015	0.003	0.066	−0.056	0.078	−0.210	0.101
Household Size													
	Small												
	Medium and/or large	−0.005	0.015	−0.035	0.025	0.004	0.010	−0.016	0.023	0.037	0.134	−0.300	0.23
Antenatal Visit													
	No visits												
	Had some visits	−0.038	0.021	−0.080	0.005	−0.005	0.017	−0.040	0.027	−0.040	0.059	−0.156	0.077
Place of Delivery													
	Home and others												
	Hospital	−0.003	0.020	−0.043	0.036	−0.018	0.012	−0.042	0.004	−0.052	0.051	−0.154	0.045
Mothers Education													
	No education												
	Primary, Secondary or Higher education	0.005	0.023	−0.040	0.051	−0.021	0.016	−0.052	0.010	−0.079	0.087	−0.250	0.095
Source of Drinking Water													
	Unprotected source												
	Protected Source	−0.006	0.022	−0.048	0.034	−0.024	0.011	−0.046	−0.002	−0.050	0.054	−0.155	0.054
Type of Toilet Facilities													
	No facility												
	Pit or flush	0.008	0.020	−0.031	0.047	0.014	0.012	−0.010	0.037	0.019	0.055	−0.085	0.125
Partners Education													
	No education												
	Primary, Secondary or Higher education	−0.027	0.020	−0.067	0.013	0.016	0.013	−0.010	0.043	−0.003	0.065	−0.132	0.119
Exposure to Mass Media													
	No Radio												
	Yes Radio	−0.007	0.018	−0.042	0.027	−0.012	0.012	−0.035	0.011	0.044	0.046	−0.043	0.134
Ever Had Vaccination													
	No												
	Yes	0.032	0.021	−0.007	0.074	−0.007	0.013	−0.033	0.019	0.025	0.051	−0.076	0.13

It is also important to highlight the seemingly counter-intuitive effect of the ever had vaccination variable in 1999 and 2010/11, which links vaccinating children with a significant increase risk to diarrhoea. In addition, type of toilet facility, exposure to mass media and partners level of education variables, had a counter-intuitive effect on childhood diarrhoea in 2010/11 and 2005/6, respectively.

**Fig. 2:**
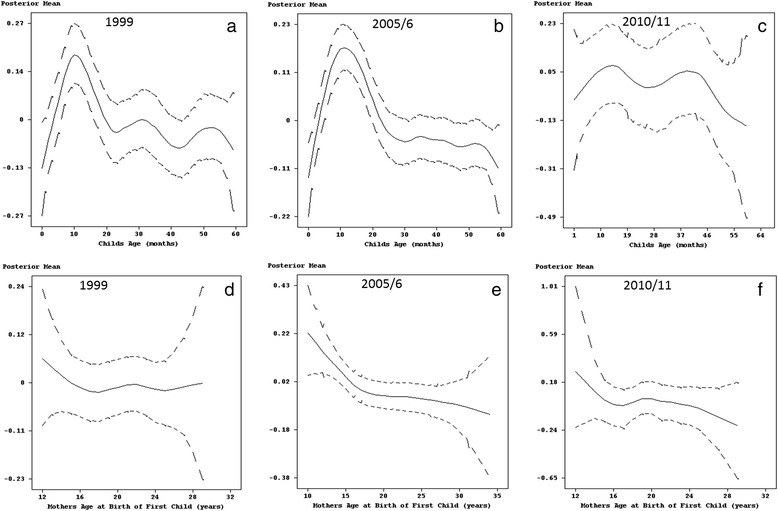
Estimated effects of child's age (panel **a, b** and **c**) and mother's age at birth of first child (panel **d, e** and **f**) on diarrhoea with 95% simultaneous cridible bands

As far as a child’s age was concerned, Fig. 2 shows a clear non-linear relationship with diarrhoea. In 1999 and 2005/6, there was a continuous and serious worsening of diarrhoeal prevalence on children up to 14 months old, followed by a sharp decreasing trend for the 14–20 months age group, with small variations in the prevalence of diarrhoea for children older than 20 months (Fig. 2a and b). However, for the survey year 2010/11 (Fig. 2c), the number of diarrhoea incidences peaked for two age groups 10–15 and 37–40 months. In both cases, this was followed by a decline in the number of incidences.

Taking into account mother’s age at the birth of the first child, diarrhoea was found across all survey years to be comparably higher in children born to young mothers (less than 16 years) than those born to middle aged women (20–35 years). For 2005/6 and 2010/11, the effect of mother’s age at the birth of first child was almost linear for mothers older than 20 years, with the posterior mean decreasing with increasing mother’s age (see Fig. 2d-f).

**Fig. 3:**
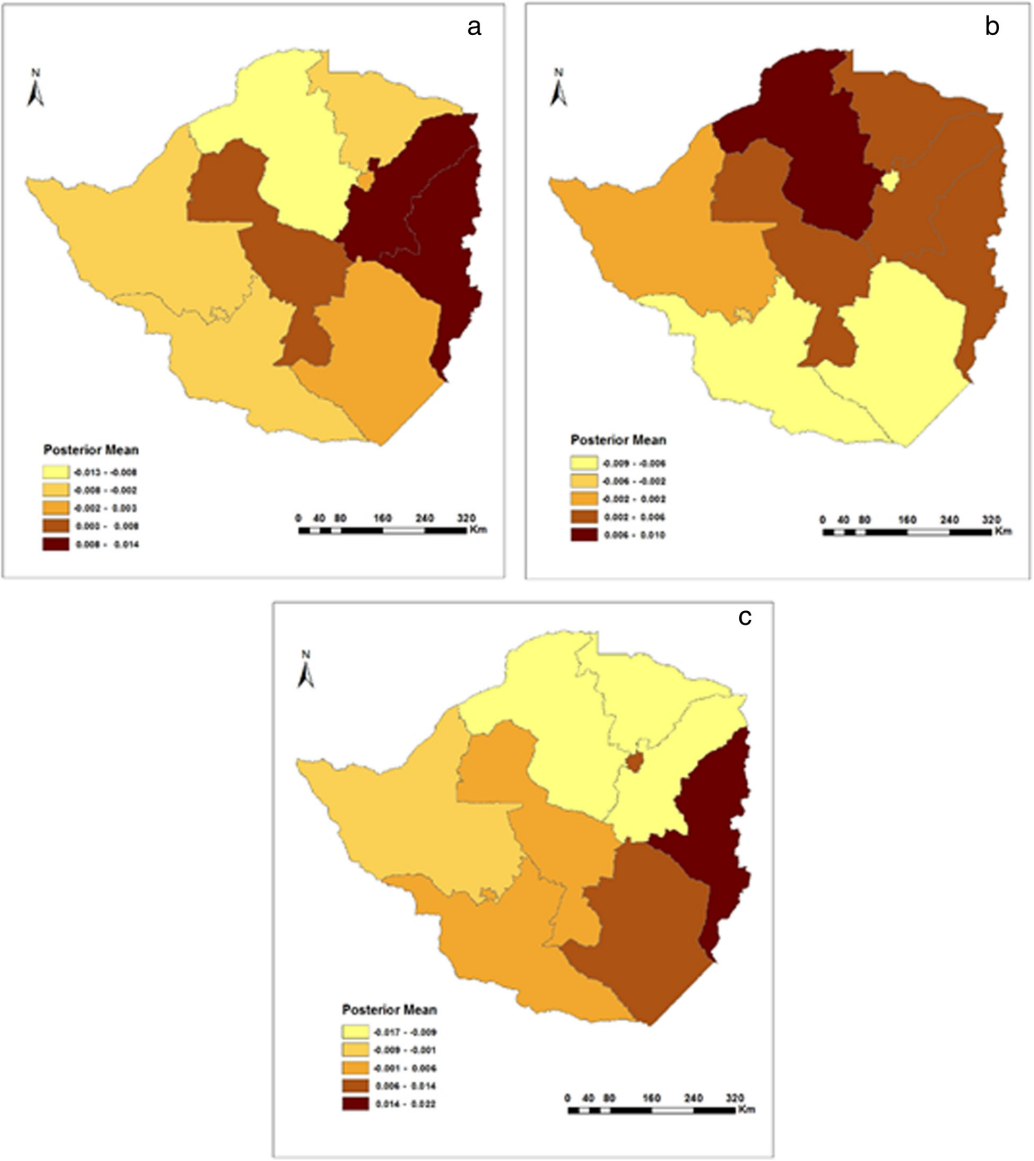
Residual spatial effects on diarrhoea in **a**) 1999, **b**) 2005/6, and **c**) 2010/11

The geographic pattern of regions in Fig. 3 shows the estimated posterior means of structured random effects on childhood diarrhoea. The evidence is such that in 1999, prevalence of diarrhoea was highest in Manicaland and Mashonaland East followed by the Midlands province (see Fig. 3a). The mainly rural based Mashonaland Central provinces experienced the lowest cases of childhood diarrhoea. In 2005/6, a large number of diarrhoeal incidences were concentrated in the north and north east parts of Zimbabwe, with the exception of Harare province, which along with Masvingo and Matabeleland South recorded the lowest number of cases (see Fig. 3b). In 2010/11, the prevalence of childhood diarrhoea also varied spatially (see Fig. 3c). Mashonaland West, Mashonaland Central and Mashonaland East, which are predominantly rural based provinces, were experiencing low incidences of childhood diarrhoea after years of recording high levels of diarrhoeal numbers in the two previous survey years.

On the other hand, Bulawayo and Harare provinces, which are mainly urban, suddenly found themselves burdened with high associations of childhood diarrhoea (Fig. 3c) than before (Fig. 3a and b). The change was however greater for Harare than it was for Bulawayo (compare Fig. 3b and c).

### Prevalence of childhood fever

The results for the fixed categorical covariates (see Table 4) indicated a negligible difference in the prevalence of fever by sex of child in both 1999 and 2005/6. However, male infants were found in 2010/11 to have a positive association with fever than their female counterparts. In addition, the survey year 1999 was characterised by the variables, preceding birth interval and type of toilet facility not showing a clear pattern on differentials by prevalence of fever. The analysis also highlights interestingly the contrasting associations between fever and marital status. Children who were raised by a single parent in 2005/6 were more likely to have fever than those raised by a married couple. However, the results for 1999 and 2010/11 offer the opposing view that children raised by a single parent were less likely to have fever than those raised by a married couple. The analysis also recognised that contrary to reviewed literature, prevalence of fever was unexpectedly higher in children who had been vaccinated, whose mothers and partner’s had attained some form of education and had access to information from mass media.

**Table 4:** Posterior Mean Estimates of the Fixed Effect Parameters for Fever

Covariate	Category	1999 Survey				2005–6 Survey				2010–11 Survey			
		Mean	SD	2.5 % Quantile	97.5 % Quantile	Mean	SD	2.5 % Quantile	97.5 % Quantile	Mean	SD	2.5 % Quantile	97.5 % Quantile
Const		0.230	0.079	0.073	0.388	0.080	0.042	0.001	0.163	0.101	0.276	−0.441	0.648
Sex of Child													
	Female												
	Male	−0.004	0.022	−0.049	0.040	0.007	0.009	−0.012	0.025	0.015	0.038	−0.057	0.087
Preceding Birth Interval													
	25 + months												
	<25 months	0.006	0.031	−0.057	0.067	−0.015	0.014	−0.041	0.011	−0.016	0.049	−0.110	0.082
Place of Residence													
	Rural												
	Urban	−0.013	0.032	−0.076	0.049	−0.011	0.017	−0.046	0.024	0.005	0.046	−0.084	0.1
Marital Status													
	Married												
	Single	−0.025	0.036	−0.097	0.045	0.041	0.014	0.013	0.068	−0.063	0.069	−0.194	0.076
Household Size													
	Small												
	Medium and/or large	−0.010	0.021	−0.052	0.031	0.006	0.009	−0.011	0.023	0.081	0.117	−0.316	0.147
Antenatal Visit													
	No visits												
	Had some visits	0.012	0.028	−0.043	0.066	0.003	0.016	−0.028	0.035	0.089	0.051	−0.010	0.186
Place of Delivery													
	Home and others												
	Hospital	−0.024	0.024	−0.072	0.024	−0.044	0.011	−0.065	−0.021	−0.093	0.043	−0.174	−0.008
Mothers Education													
	No education												
	Primary, Secondary or Higher education	0.046	0.032	−0.017	0.109	−0.005	0.014	−0.034	0.023	0.056	0.072	−0.089	0.196
Source of Drinking Water													
	Unprotected source												
	Protected Source	−0.019	0.028	−0.075	0.037	0.010	0.011	−0.011	0.031	0.015	0.045	−0.077	0.103
Type of Toilet Facilities													
	No facility												
	Pit or flush	−0.006	0.026	−0.056	0.043	−0.005	0.011	−0.026	0.017	−0.023	0.047	−0.116	0.07
Partners Education													
	No education												
	Primary, Secondary or Higher education	−0.022	0.027	−0.075	0.030	−0.002	0.013	−0.027	0.023	0.053	0.056	−0.055	0.164
Exposure to Mass Media													
	No Radio												
	Yes Radio	−0.012	0.023	−0.057	0.034	−0.001	0.011	−0.021	0.022	0.081	0.037	0.008	0.152
Ever Had Vaccination													
	No												
	Yes	0.023	0.027	−0.031	0.075	0.003	0.013	−0.021	0.028	0.036	0.043	−0.051	0.123

**Fig. 4:**
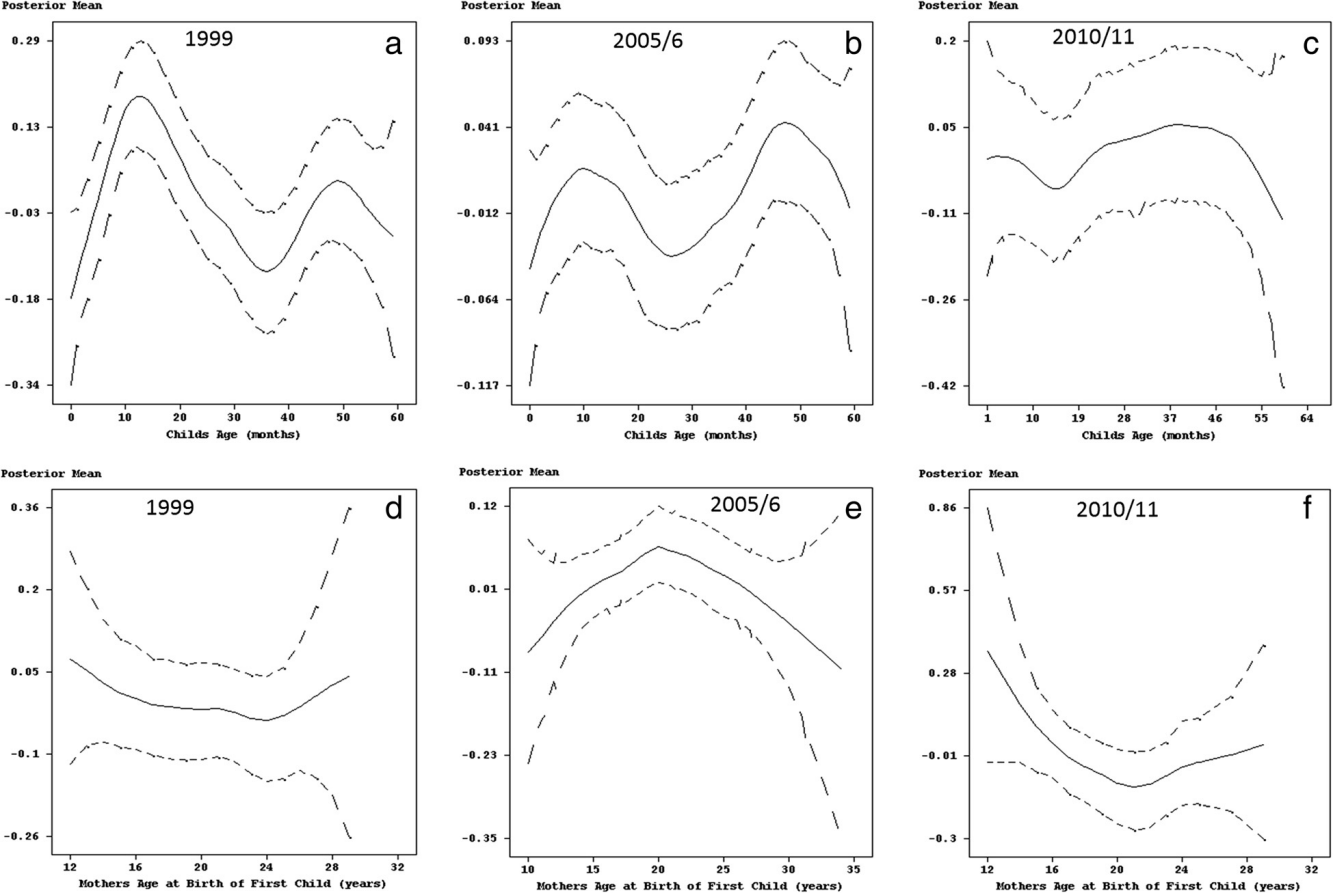
Estimated effects of child's age (panel **a**, **b** and **c**) and mother's age at birth of first child (panel **d**, **e** and **f**) on fever with 95% simultaneous cridible bands

With regards to mother’s age at birth of first child and child’s age, there was clearly a non-linear relationship with childhood fever (see Fig. 4). The number of cases of fever in 1999 and 2005/6 was highest among infants, reaching a peak for children approximately 10–14 months old. This was followed by a decline in the number of cases for the age groups 14–36 months and 10–25 months for the years 1999 and 2005/6, respectively. In both survey years, there was an increase in the number of cases thereafter, but was soon followed by a steady decline in children older than 48 months (Fig. 4a and b). On the other hand, in 2010/11 older children of the age group 26–40 months were more likely to be sick with fever, followed by infants aged 1–6 months (Fig. 4c).

Furthermore, the results for 1999 highlighted a considerably higher number of cases in children of young mothers (less than 14 years old) and those who gave birth to their first child after their 24th birthday (Fig. 4d). In 2005/6, fever was more prevalent in children born to young mothers and reached its peak for mothers 20 years old, with the number of cases declining thereafter for middle aged mothers (Fig. 4e). As for 2010/11, childhood fever was considerably higher in children of young mother’s. This was followed by a decline with children born to mother’s aged 18 years recording the lowest number of cases (Fig. 4f). Thereafter, the number of incidences increased steadily as mothers age increased.

**Fig. 5:**
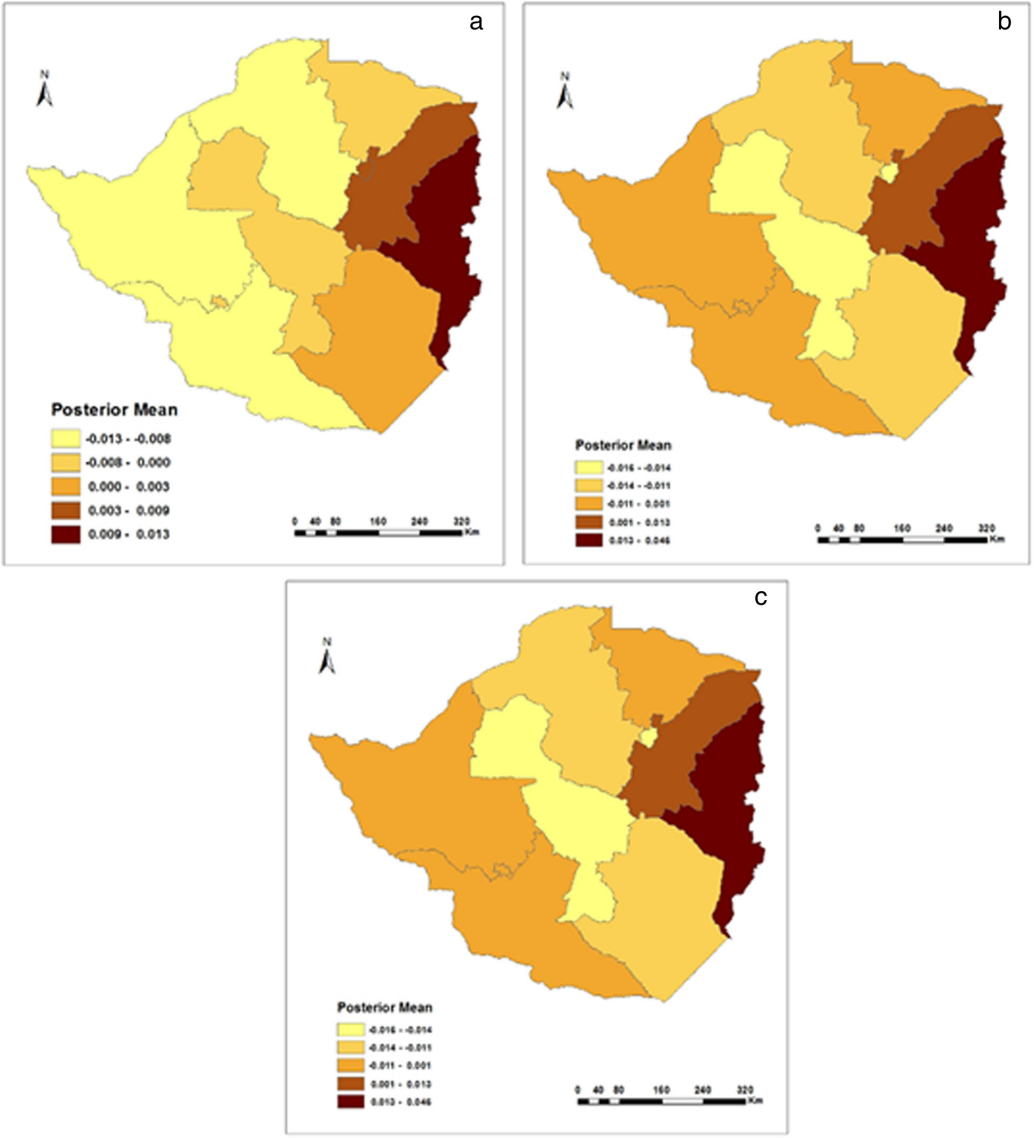
Residual spatial effects on fever in **a**) 1999, **b**) 2005/6, and **c**) 2010/11

In addition, Fig. 5 shows how childhood fever varied by province between 1999 and 2010/11. In 1999, there was an increase in the prevalence of childhood fever as one moved from provinces in the west to those east of Zimbabwe (see Fig. 5a). As such, the Matabeleland North and South provinces had the lowest association with childhood fever, whilst at the extreme end of the scale was the Manicaland province. Geographically, 2005/6 saw the highest prevalence of childhood fever shift to provinces in the north (Mashonaland West and Central) and west (Bulawayo, Matebeleland North and South) of Zimbabwe (see Fig. 5b). In 2010/11, the prevalence of fever maintained the same status quo as in 2005/6 (Fig. 5c).

### Prevalence of childhood cough

In the 1999, 2005/6 and 2010/11 ZDHS’s, the prevalence of childhood cough was collected based on the mother’s perception of illness and was not validated by medical personnel. Mothers were asked whether their children under the age of five years had been ill with a cough accompanied by short, rapid breathing in the two weeks preceding the survey. The 1999 ZDHS data shows that contrary to relevant literature, households within which children had been vaccinated, had access to either pit of flush toilets, with mother’s who had attained some form of education and had a preceding birth interval of less than 25 months had a high significant effect on childhood cough. On the other hand children born in hospitals, raised in an urban setting and had access to a protected water source were linked with a low risk of cough. The fixed covariates for 2005/6 shown in Table 5 point to a significant lower risk of cough associated with children born in hospitals and those vaccinated as part of the Zimbabwe’s public health strategy for the prevention of vaccine-preventable diseases. However, single parenting, making antenatal visits and raising a child in a medium or large family had a positive association with childhood cough. Also evident in this survey year, is the little or no significant association between childhood cough and the covariates; sex of child, preceding birth interval, place of residence, mother’s and partner’s level of education, type of water source, type of toilet facility and exposure to mass media.

**Table 5:** Posterior Mean Estimates of the Fixed Effect Parameters for Cough

Covariate	Category	1999 Survey				2005-6 Survey				2010-11 Survey			
		Mean	SD	2.5 % Quantile	97.5 % Quantile	Mean	SD	2.5 % Quantile	97.5 % Quantile	Mean	SD	2.5 % Quantile	97.5 % Quantile
Const		0.328	0.088	0.156	0.504	0.163	0.060	0.044	0.282	0.210	0.269	−0.316	0.936
Sex of Child													
	Female												
	Male	0.004	0.023	−0.042	0.049	−0.007	0.013	−0.034	0.018	0.015	0.037	−0.058	0.087
Preceding Birth Interval													
	25 + months												
	<25 months	0.031	0.034	−0.036	0.096	−0.004	0.019	−0.041	0.033	−0.008	0.048	−0.102	0.089
Place of Residence													
	Rural												
	Urban	−0.047	0.035	−0.115	0.024	−0.006	0.026	−0.058	0.042	−0.019	0.046	−0.109	0.072
Marital Status													
	Married												
	Single	0.003	0.041	−0.074	0.086	0.016	0.020	−0.023	0.054	0.072	0.068	−0.058	0.21
Household Size													
	Small												
	Medium and/or large	−0.007	0.023	−0.052	0.038	0.014	0.013	−0.010	0.040	0.054	0.117	−0.278	0.178
Antenatal Visit													
	No visits												
	Had some visits	0.007	0.030	−0.053	0.067	0.018	0.021	−0.024	0.059	0.124	0.049	0.026	0.218
Place of Delivery													
	Home and others												
	Hospital	−0.020	0.028	−0.075	0.034	−0.061	0.015	−0.091	−0.031	−0.086	0.043	−0.169	−0.001
Mothers Education													
	No education												
	Primary, Secondary or Higher education	0.046	0.035	−0.021	0.119	0.008	0.020	−0.031	0.048	0.096	0.075	−0.058	0.244
Source of Drinking Water													
	Unprotected source												
	Protected Source	−0.039	0.031	−0.100	0.022	−0.005	0.015	−0.033	0.024	0.106	0.045	0.019	0.194
Type of Toilet Facilities													
	No facility												
	Pit or flush	0.049	0.028	−0.004	0.102	0.009	0.015	−0.020	0.041	−0.138	0.047	−0.229	−0.047
Partners Education													
	No education												
	Primary, Secondary or Higher education	−0.008	0.030	−0.063	0.050	−0.001	0.018	−0.033	0.034	−0.011	0.056	−0.121	0.095
Exposure to Mass Media													
	No Radio												
	Yes Radio	0.007	0.026	−0.044	0.059	0.001	0.015	−0.028	0.031	0.056	0.039	−0.018	0.129
Ever Had Vaccination													
	No												
	Yes	0.060	0.029	0.003	0.119	−0.010	0.018	−0.046	0.024	−0.066	0.045	−0.158	0.02

As for 2010/11, results from this survey year indicate that male infants were at a greater risk of cough than their female counterparts. Children living in an urban setting were at a lesser risk of cough than those living in rural areas. There was also a significant high association of cough with children raised by a single parent, who had access to a protected water source, born of mother’s who had acquired some form of education, had access to a variety of health information sources and had made one or more antenatal visits during pregnancy. On the other hand and in line with the relevant literature children who had been vaccinated, were born in a hospital and had access to clean pit or flush toilet facilities were associated with significant low risk of cough

**Fig. 6:**
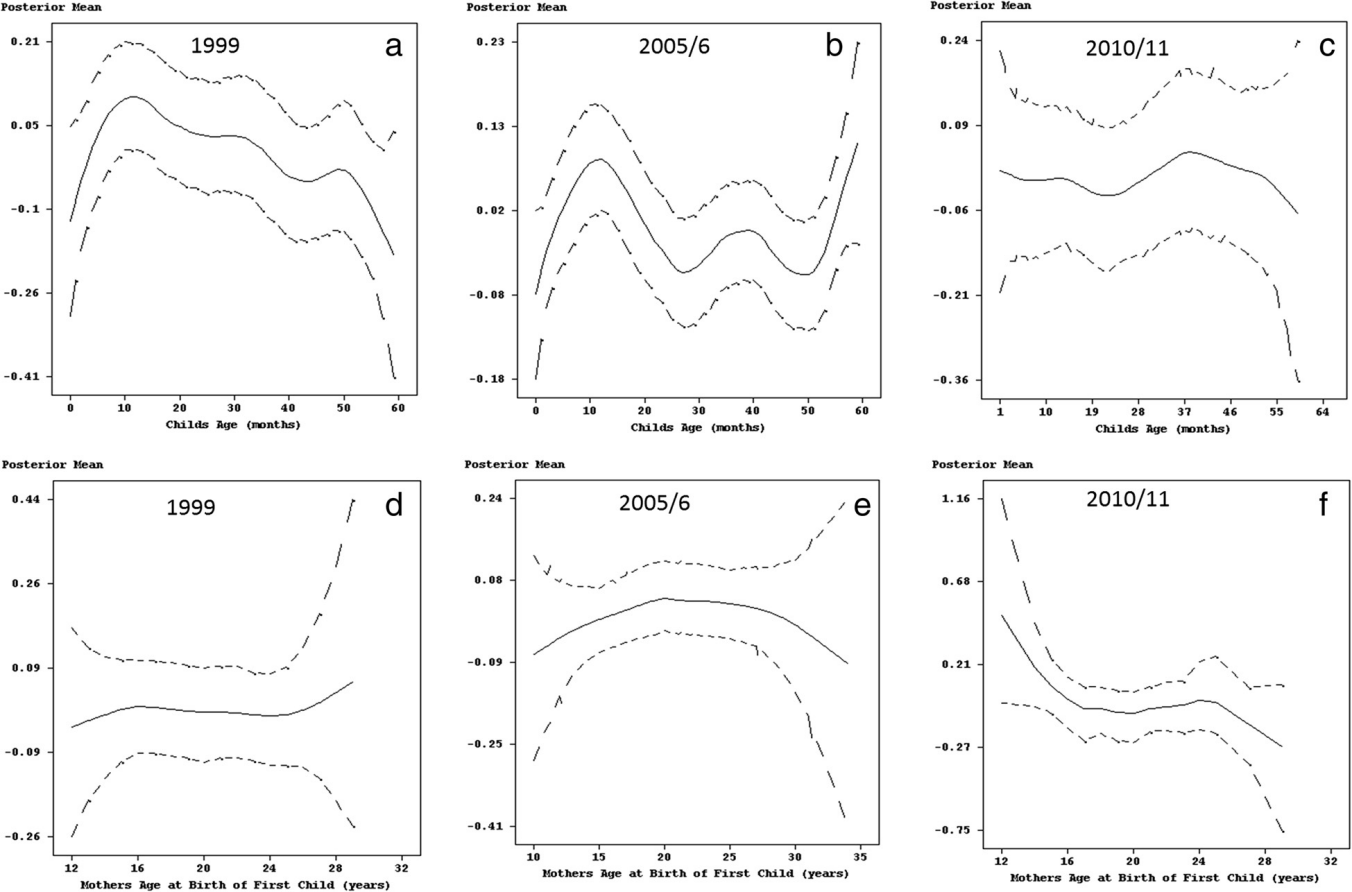
Estimated effects of child's age (panel **a**, **b** and **c**) and mother's age at birth of first child (panel **d**, **e** and **f**) on cough with 95% simultaneous cridible bands

The prevalence of cough also varied with child’s age. In 1999, cases of childhood cough reached a peak at approximately 10 months of age, then fell thereafter (see Fig. 6a). The 2005/6 data showed a continuous increase in the number of children up to 12 months old who had been affected by cough. Fig. 6b also shows how after a decline, childhood cough in 2005–6 increased for children between 25 and 40 months old. Although this was followed by another decline this was short-lived as highlighted by another increase in childhood cough for children older than 50 months. In contrast to the previous survey year, childhood cough in 2010/11 followed a declining trend for children up to 22 months old. Thereafter, the number of cases increased for the age group 25–40 months, finishing off with a steady decline for older children (see Fig. 6c).

Furthermore, as far as mother’s age is concerned, childhood cough in 1999 showed a steady increase in children of young mothers up to 16 years old, declined slightly for children of middle aged mothers (16–25 years), and had the largest effect in children born to mothers older than 25 years (see Fig. 6d). The association between childhood cough and mother’s age at birth of first child in 2005/6 was characterised by an increase in the number of cases in children born to mothers who had given birth to their first child aged 20 years or less followed by a decline for children of mothers who had given birth to their first child in middle and/or older age (Fig. 6e). In addition, the prevalence of cough in 2010/11 was highest for children born to mothers who had their first birth at a young age but also showed a decline in the number of cases for mothers up to 18 years old. This was followed by a small increase for children born to mothers who had their first birth aged 20–25 years old and then another decline thereafter (see Fig. 6f).

**Fig. 7:**
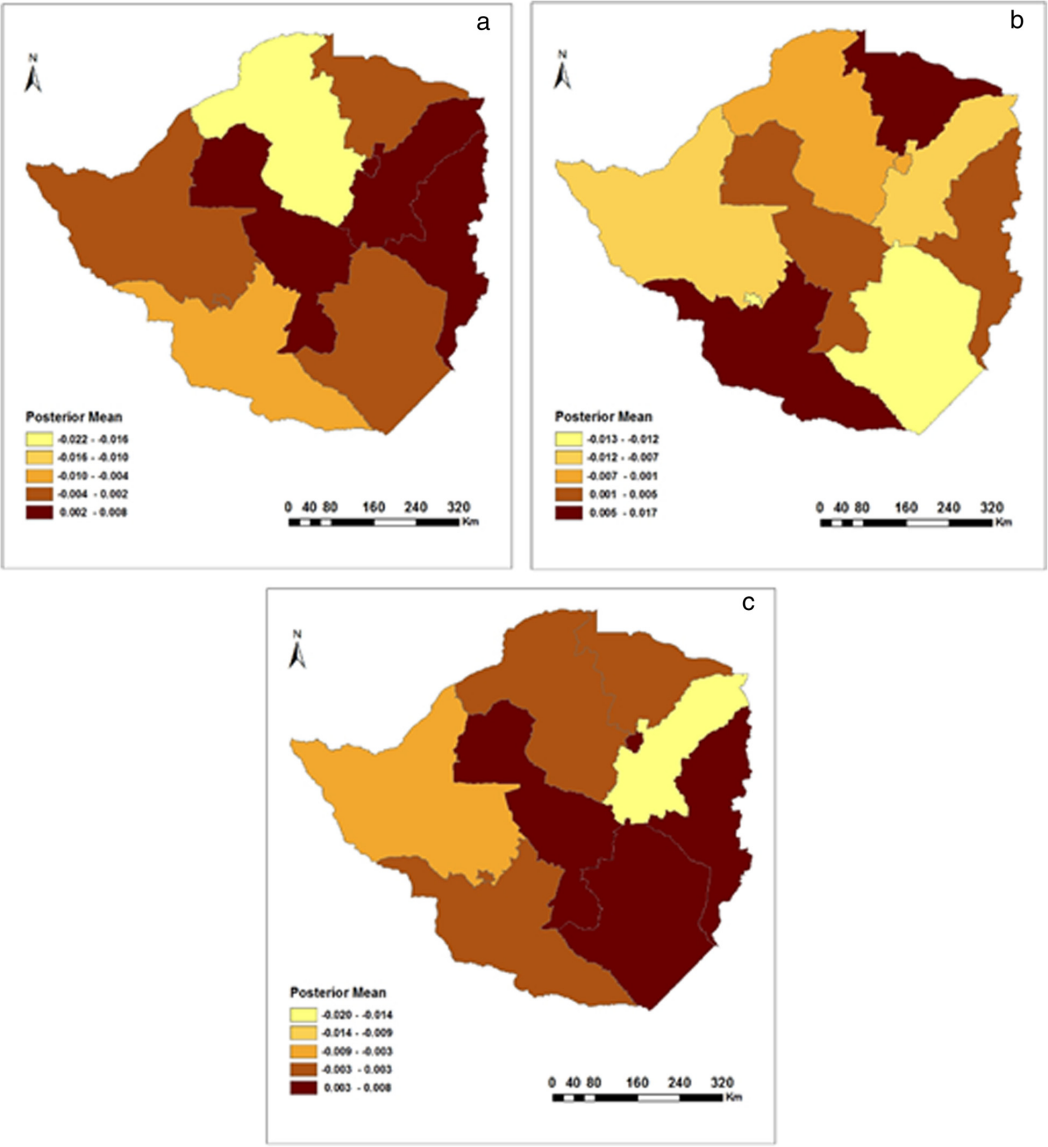
Residual spatial effects on cough in **a**) 1999, **b**) 2005/6, and **c**) 2010/11

With regards to spatial effects, urban–rural and provincial differences had a substantial association with childhood cough. 1n 1999, children in rural areas were much more likely than urban children to have been ill with cough in the past two weeks before the survey. This is supported by the fact that spatial effects (see Fig. 7a) highlight the highest number cases to have occurred in rural based Manicaland, Mashonaland East, Masvingo, Mashonaland West and Matabeleland North provinces.

Geographically, the prevalence of childhood cough in 2005/6 was worse in Mashonaland West and Matabeleland South when compared to the 1999 survey year. Although the number of cases declined in other provinces, childhood cough still remained a major problem in Manicaland and Midlands provinces. Urban based Bulawayo and rural-based Masvingo provinces recorded the lowest number of cases in childhood cough (see Fig. 7b). Finally, in 2010/11 a large portion of Zimbabwe experienced a high number of cases of childhood cough with the Manicaland, Harare, Masvingo and Midlands provinces carrying the majority of the burden (see Fig. 7c). Not far behind were the rural based Mashonaland West and Central as well as Matabeleland South provinces. In contrast, Mashonaland East experienced the lowest number of cases when compared to the previous survey years.

### Sensitivity analysis

In some situations, the estimation results of a full Bayesian semi-parametric regression model depends on the choice of hyper-parameters, for example, the parameters *a* and *b* defining the inverse gamma (IG) prior of the variances of nonparametric and spatial effects. Hence, it is often recommended to check how sensitive the results are with changes in the hyper-parameters. In this study, the Markov Chain Monte Carlo (MCMC) simulations were rerun using Model 3 for simplicity, to investigate the sensitivity of the results to different choices of hyper-parameters. In particular, the following alternatives of priors were investigated:

$$ IG\left(a=001,b=0.01\right),\kern0.24em IG\left(a=0.5,b=0.0005\right)\kern0.5em  and\;IG\left(a=1,b=0.0005\right) $$

The first alternative and the standard option *IG*(*a* = 0.001, *b* = 0.001) are commonly used choices for the variances of random effects. The second and third alternatives were suggested by Kelsall & Wakefield [22] and Besag & Kooperberg [23], respectively. Results of the sensitivity analysis on the choice of hyper-parameters *a* and *b* are shown in Table 6. Only results for one model for each dataset are shown.

**Table 6:** Summary of the sensitivity analysis of the choice of hyper-parameters

	Using data on childhood diarrhoea from the 1999 survey				Using data on childhood cough from the 2005/6 survey				Using data on childhood fever from the 2010/11 survey			
	a = 0.001	a = 0.01	a = 0.5	a = 1	a = 0.001	a = 0.01	a = 0.5	a = 1	a = 0.001	a = 0.01	a = 0.5	a = 1
	b = 0.001	b = 0.01	b = 0.0005	b = 0.005	b = 0.001	b = 0.01	b = 0.0005	b = 0.005	b = 0.001	b = 0.01	b = 0.0005	b = 0.005
Spatial effects												
*f*_*str*_(*s*), *τ*_*str*_^2^	0.005	0.008	0.002	0.002	0.011	0.017	0.004	0.003	0.019	0.023	0.005	0.004
*f*_*unstr*_(*s*), *τ*_*unstr*_^2^	0.002	0.004	0.001	0.001	0.005	0.006	0.003	0.003	0.006	0.009	0.003	0.003
Smooth functions												
*f*_1_(*Childs age*), *τ*_1_^2^	0.000	0.000	0.000	0.000	0.000	0.000	0.001	0.000	0.000	0.001	0.000	0.000
*f*_2_(*Mothers age*), *τ*_2_^2^	0.001	0.002	0.001	0.001	0.001	0.002	0.000	0.000	0.012	0.018	0.006	0.003

Re-running MCMC simulations based on the four choices of hyper-parameters yielded minor differences between the variance parameters for the spatial effects and non-linear functions. This indicates that the models used in this study were less sensitive to the choice of hyper-parameters, thus were appropriate for all the analysis.

## Discussion

### Fixed categorical effects

The results suggest a complex and highly correlated array of determinants that have influenced childhood diseases in Zimbabwe since the 1999 ZDHS. Until recently, sex of child had little or no significant association with childhood diseases. However, as of 2010, a higher proportion of male than female children within a given province had a significant association with childhood cough, fever and diarrhoea. One possible theory to explain this sex imbalance on childhood diseases originates from a biological point of view, which suggests that male children have a weaker immune system than their female counterparts, which increases their susceptibility to infectious diseases [24].

In addition to the gender-related factors, urban children had a significantly lower association with cough, fever and diarrhoea than rural children across all the survey years with the exception of diarrhoea in 2010. Smith and Kohn [25] and Kandala et *al.* [8] have attributed this to the fact that urban children are twice more likely to be immunized and have greater access than their rural counterparts to better basic health-care infrastructure, clean water, sanitation, and education. In 1999, 81% of urban children in Zimbabwe were vaccinated compared to 72% in rural areas. Children in urban areas had better vaccination coverage (58%) in 2005/6 than their counterparts in rural areas (50%). Similarly, in 2010/11, children in urban areas were more likely than rural children to have received all basic vaccinations (70% compared with 62%). Smith and Kohn [25] also argue that urban areas have higher population densities, making it easier to share information and resources on child health care. In contrast, the majority of rural areas in Zimbabwe are sparsely populated areas with the least adequate infrastructure thus compromising children access to basic health care services. The high association between diarrhoea and urban areas in 2010/11 can be linked to water shortages and insufficient sewerage systems, which continue to plague Zimbabwe’s urban areas. Compounded by an increase in population due to rural–urban migration as people pursue better livelihood (economic and social benefits), Zimbabwe’s sewage and wastewater treatment plants continue to fail, and in most drastic situations council officials often redirect untreated human waste into main water sources thus an upsurge in cases of diarrhoea and dysentery [26–28]. In addition, although children in these urban areas enjoy greater access to health services, for those who live in most densely populated areas (Chitungwiza, Mbare, Mufakose, Highfield, Epworth etc.), basic health care has been compromised [29]. This is likely a reflection of overcrowded or slum conditions where, similar to remote rural areas, health care services have become inadequate.

In Zimbabwe, as in most less-developed countries, household size plays a major role in the quality of health received by the individuals of a particular family. The household size was significantly associated with the prevalence of childhood diarrhoea, fever and cough. Having a medium and/or large family of six or more members means that contact between potential infectives and susceptibles will be of a higher order than in smaller households (less than six family members). In addition, in large families, the caretakers will have less time to look after individual children; hence, they may end up receiving poorer quality of care. As a result, in a high-risk environment, they are likely to come into contact with more pathogens, bacteria or virus that cause diarrhoea, fever or cough.

Associated with the household environment is sanitation, which in this study was measured by factors such as source of drinking water and toilet facilities. According to Esret and Habicht [30] and Merrick [31], sanitation is an important determinant covarying more with older children’s chances of contracting diseases than with infants. For children, their well-being is highly dependent on both the quality and the availability of water and sanitation facilities. Results from this study show that sanitation can have two different effects on childhood diseases. First, the general results were intuitive: the provision of piped drinking water and pit or flush toilets to households had a low association with childhood diseases. Access to piped water, using proper toilets and hand washing – preferably with soap – prevents the transfer of bacteria, viruses and parasites which otherwise contaminate water sources, soil and food. This contamination is a major cause of diarrhoea, the second biggest killer of children in developing countries such as Zimbabwe. In addition, improved sanitation reduces the chances of older children, who are often unsupervised or afforded some degree of freedom, coming into contact with excreta or contaminated water as they explore their immediate environment. This is despite the fact that in Zimbabwe there has been a slight decline in the proportion of the population which has access to improved sanitation and water facilities (73% in 2009 compared to 76% in 2005/6). This proportion is biased towards the urban areas. For example, it was reported that in 2009, the province of Harare (mainly urban) had improved sanitation facilities as high as 97%, while rural based Matabeleland North use of such facilities was as low as 30%. Secondly, the results also pointed towards the fact that the existence of a flush or pit latrine and piped water increased the chances of childhood diseases. This result, although counterintuitive at first, might signal poor quality of sanitation and water facilities. Another school of thought is that since a large part of Zimbabwe’s sanitation and water sources are shared, this counterintuitive effect may be linked to the negative externalities involved in shared water sources and sanitation facilities. Even if ownership is established and one has to pay to gain access, households simply do not have an incentive to keep the place clean, which is a classic problem with common goods. For operators, such as local authorities, constant monitoring is an extra cost that they are not willing to incur or can afford. Hence, it is this specific channel that is potentially a source of pathogens.

Furthermore, the surveys also revealed contradicting associations between parental education and childhood diseases. Contrary to a large portion of the relevant literature, higher education in mothers increased the prevalence of cough and fever in 1999 as well as in 2010. In addition, having an educated partner also increased the prevalence of diarrhoea in 2006 and fever in 2010. One possible explanation could be that well-educated parents may be unable to reduce the risk of exposure of their children due to factors beyond their control, such as contaminated community environments or lack of water. A possible explanation to this phenomenon is unknown and the author suggests further investigation. However, well-educated parents greater knowledge of nutrition, hygiene and other practices related to childcare could have contributed to the low prevalence of diarrhoea in 2006 and 2010. According to Boerma [32], parental education is most needed to counteract childhood diseases in older children than in infants, presumably because older children are more reliant on health facilities, clean hygiene practices, and quantity and variety of solid food – factors that better educated parents are more likely to seek out and gain access to.

Proximate determinants of childhood diseases also include maternal risk factors, which are more related to early infant diseases because of their association with premature and low birth weight infants and delivery complications. One of the most important maternal factors found to be related to childhood diseases in this study is the pace of childhood bearing. In particular, short preceding birth intervals were found to increase an infant’s risk to childhood diarrhoea, fever and cough because the mother’s nutritional reserves have not fully recovered from the previous birth. It is also vital to mention that these short birth intervals in Zimbabwean women may also have affected the older children as well by creating competition between young siblings for the mother’s resources such as breast milk, attention and other kinds of care such as bathing, cleaning and clothing.

In addition to preceding birth interval, antenatal care is another maternal factor found in this study to be related to childhood diseases. The antenatal care period clearly presents opportunities in the continuum of care process for reaching pregnant women with interventions that are vital to their health and well-being and that of their infants. Antenatal care coverage has remained generally high in Zimbabwe. Nationally this was reported in MIMS as 93% in 2009, which is a slight decline from 95% in 2006 [33]. It is likely that hospitals will use the antenatal visits to provide mothers with advice and administer professional health care in order to reduce the risk of diarrhoea, cough and fever in newly born babies. Hence, this study found children of mothers who made frequent antenatal visits during their pregnancy had a low association with childhood diseases. In contrast, the results also indicated a negative association between childhood diseases and children of mothers who received antenatal care during their pregnancy. The author can only speculate that poor quality of services is the main cause of this counterintuitive effect, as high patient volume and limited resources combine to constrain service provision, however further investigation is necessary. In the case of Zimbabwe, government sponsored hospitals, which are relied upon by 90% of the mothers have now themselves become a threat to patients. A case in point is the Harare Central Hospital, one of the country’s largest referral hospitals, which is now in a shambolic state of leaking roofs, peeling floors and cracked walls. These have become breeding grounds of bacteria and pathogens, exposing patients including pregnant mothers and those in labour to more diseases. In addition, due to the brain drain which has affected Zimbabwe’s healthy system, pregnant mothers have to contend with being treated by unqualified staff and not spending enough time with their doctors due to a high patient to doctor ratio. In such cases, symptoms of diseases that may affect a mother and her child may be missed even though records will show that they have had antenatal care throughout their pregnancy.

Considering the advantages of antenatal care mentioned above and the high national coverage, the assumption was that this would lead to a high percentage of babies born in Zimbabwe to be delivered in a health facility. However, contrary to this widely held assumption, deliveries at health facilities have actually decreased substantially over the last decade, although antenatal visits have increased. In 1999, 83% of deliveries in Zimbabwe occurred in health institutions. According to ZDHS in 2005/6 [33], 68% were institutional deliveries, and in 2009, only 61% of deliveries occurred in health institutions. This marked imbalance between antenatal attendance and corresponding deliveries at health facilities shows a consistent decline in institutional deliveries over the years, compromising safety of both mother and baby during childbirth. However, in situations where babies have been delivered in a health facility (mainly private hospitals), the advantages have been numerous. Proper medical attention and hygienic conditions during delivery can reduce the risks of complications and infections that can cause morbidity and mortality to either the mother or the baby. In addition, mothers can take advantage of the advice they receive from doctors and nurses before they leave the hospital on how best to protect their children from contracting diseases at such at an early age when they are most vulnerable. Hence, this study found a low association between childhood diseases and children delivered in a hospital environment.

According to the literature, vaccination of children from birth helps to eradicate and prevent various diseases. There is certainly strong evidence that vaccination significantly reduces childhood diseases such as polio, measles, meningitis and many others. The belief in vaccinations is such that it is now compulsory in most countries for every child to be vaccinated. Hence, it was unexpected that results from this study showed a high association between childhood diseases and vaccination. One possible reason for this counterintuitive effect could be the high percentage of missing values associated with this variable could have affected the results. Another possible reason could be that children in Zimbabwe are usually vaccinated against polio, measles, tuberculosis, diphtheria, whooping cough and tetanus instead of diarrhoea included in this study.

### Non-linear effects

In general, the results show that child’s age has a non-linear relationship with all three diseases. The prevalence was highest between the ages of 6 and 15 months. Beyond 15 months, incidences of childhood diseases steadily declined with age. This period is when most children are weaned. According to the World Health Organization (WHO) a child’s immune system between the ages of 6 and 15 months is not sufficiently developed to protect him or her from first exposure to enteric pathogens via contaminated complementary and/or weaning food or contact with the environment when crawling. In addition, because of poverty, insufficient household sanitary conditions, which have been affecting Zimbabwe since the late 1990’s, mothers have been finding it difficult to feed infants with enough clean breast milk so that mixed low-quality foods or contaminated water were used instead [34]. The situation is compounded by the fact that most families in Zimbabwe live below the poverty datum line and find it difficult to afford high-quality foods to boost their children immune system at this early age. However, as the child grows, the immune system develops, becomes stronger and is able to fight off diseases, which is reflected by the gradual decline of the effect of age after 15 months. This pattern resembles those found in many studies of sub-Saharan Africa [8, 6, 11]. Furthermore, there were also survey years (cough in 2005/06, cough in 2010/11, diarrhoea in 2010/11, fever 2005/06 and fever in 2010/11) were older children (older than 37 months) had similar or higher incidence rates of diseases to the infants aged 6–15 months. This is unusual, because older children are considered to be more resistant to pathogens, bacteria or virus, and their greater awareness of the environment is likely to reduce exposure. On the other hand, children of this age have considerable independence, are highly mobile, and play unsupervised within the community. Hence, in an environment where there is a large number of infectives, this age group is likely to have a high degree of exposure to childhood disease pathogens.

The approximate negative linear relationship between age of mothers at first birth of 20–40 years and the three diseases suggest that a child’s association with diarrhoea, cough and fever declines as their mothers get older. In Zimbabwe, like in most sub-Saharan African countries, where women give birth to their first child at around 20 years of age, first births are associated with very young mothers. According to Zenger [35], these women’s children carry a higher risk of diseases because young, first parity mothers may not have reached their full physical and reproductive maturity. However, a more detailed analysis of trends in age at first birth does reveal a decline in early childbearing. Whereas on average about 24% of women aged 35–39 had a birth at age 18, only 21% of women currently aged 20–24 had their first birth at age 18. This slow but steady trend reflects positively on efforts to keep girls and women in school through more advanced levels to improve their social and economic status. Furthermore, findings regarding children of older mothers and mothers of high parity vary more, but because of the increased risk of delivering a genetically impaired infant later in life, these children are also likely to carry higher risk of diseases as highlighted in Figs. 4 and 7 [36].

### Spatial effects

The structured spatial effects included within the models used in this study are often surrogate measures of unobserved spatially correlated risk factors of childhood diseases. The results show clear evidence of significant high prevalence of childhood diseases in Mashonaland than in Matebeleland provinces. This may have been influenced by the population densities of the respective provinces. Population density is lower in Matabeleland than in Mashonaland provinces. By having unequal population densities, bias could have been introduced in the results in that a smaller sample of data was collected in one province than in another hence more cases of childhood diseases were recorded in Mashonaland than in Matabeleland provinces. In addition, the unavailability or inaccessibility of health facilities can be an explanation for the spatial distribution of childhood diseases as they vary by province. Even the most powerful diagnostic tests, drugs and vaccines have little public health impact if they do not reach the people that need them the most. Children in predominantly rural based provinces (Matabeleland North, Matabeleland South, Mashonaland West, and Mashonaland Central) face problems of accessibility, including distance, long travel times to health facilities, and scarce public transport [37–39]. Even in those provinces with health facilities, they are less likely to have well trained staff or be stocked with appropriate drugs and equipment. Religion and cultural beliefs in these provinces also act as a barrier and affects health seeking behaviour of parents, which in turn affects their children [40, 41] (Shoko, 2007; van den Heuvel *et al.,* 1999). Even where health services are available (Harare, Bulawayo, for example) issues related to affordability are major obstacles [42–44]. The cost of seeking child care (such as user fees, and transport costs) may delay or prevent poor households from accessing health services hence an increase in childhood diseases in these provinces.

Furthermore, the high prevalence of childhood cough in rural based Manicaland, Midlands and Mashonaland West provinces can be linked to unclean cooking fuels such as firewood, charcoal, crop residues or dung and coal. These fuels emit chemicals such as carbon monoxide, nitrogen dioxide, benzene or formalde-hyde, which are known hazards that affect the respiratory system and cause coughing among children and adults as they are exposed to this form of indoor air pollution.

Moreover, climate (including rainfall, temperature and soil) is another factor that can be used to explain the spatial distribution of childhood diseases. It is of potential interest because it incorporates factors affecting agricultural production and disease transmission (through vector, water and airborne mechanisms). High incidences of childhood diarrhoea may be linked with seasonal rainfall in Manicaland, Midlands and Harare provinces. During dry periods, pathogens accumulate in the environment and when heavy rain falls it can act as a mode of transmission for the pathogens. Children ingest these pathogens when they consume untreated water thus an increase in diarrhoeal incidences. However, in some provinces, during wet periods the pathogens may be regularly flushed from the environment and heavy rainfall can further dilute their concentration, which means fewer people will be exposed. This may account for the low diarrhoea incidences. These observations are the author’s alone and future research is recommended to further substantiate these claims.

### Advantages and limitations of the study

Unlike classical modelling approaches, the data-driven methodological concept used in this analysis provides tools for handling both linear and non-linear covariates within the same framework. Such modelling flexibility is useful to establish a better epidemiological relationship that exists between childhood diseases and risk factors. This study also demonstrates how spatial analysis may reveal some salient features of childhood diseases, which may be overlooked by the classical regression models (Model M0). In addition, since the ZDHS data is based on a random sample of provinces, the structured component used in this study allows one to “borrow strength” from information of adjacent neighbours to cope with the sample variation of the province effect and obtain estimates for areas that may have inadequate sample sizes or be unsampled [8]. Moreover, even though the methodology is mathematically intensive, the availability of the public domain software, BayesX, and relevant literature provides opportunities for non-mathematicians or non-programmers to utilize these methods.

On the other hand, one of the limitations of this study is based on the Deviance Information Criterion (DIC). Although the DIC is now widely used for model choice selection in complex Bayesian models, its usage is at least debatable [21]. The DIC measures only the relative goodness of fit among a collection of models. It does not provide information on the adequacy of the model. Furthermore, the definition of childhood diseases used in this study was broad; no distinction was made between acute and persistent diarrhoea, cough or fever; and related to this duration of episodes was not considered. In future research, more specific definitions of cough, fever and diarrhoea, especially if causative pathogens were isolated, might give a more precise picture of how these childhood diseases are transmitted and the risk factors associated with them. It was also felt that the samples for each survey year were small meaning that only relatively large differences in childhood diseases were statistically significant. Future research should combine the survey data. By pooling the data, a larger sample is obtained for each province, thus increasing the statistical power of the analysis. Finally, sampling data at the Enumeration Area (EA) level for a country such as Zimbabwe is inadequate. Although costly, future ZDHS’s should record data at the district level in order to gain a better understanding of childhood diseases at such a small scale.

## Conclusion

This study applies a Bayesian semi-parametric modelling approach to analyse proximate determinants of childhood diarrhoea, fever and cough, using data from the Demographic and Health Surveys (DHS). Such a flexible modelling approach allows joint analysis of nonlinear and fixed determinants either at group or individual level, as well as observed and unobserved determinants, which may exhibit spatial variation. Results from the models reveal that childhood diseases are associated with sex of child, place of residence, place of delivery, sanitation, antenatal care, vaccination, preceding birth interval, marital status, household size, parents level of education, child’s age and mother’s age at birth of first child. Depending on the data, the association can be either intuitive or counterintuitive. Furthermore, childhood diseases in Zimbabwe exhibit some spatial variation, which can be linked to factors such as climate, population density, availability and accessibility of health facilities. These findings should serve as novel information to help health planners and policy makers in making effective decisions about childhood diarrhoea, cough and fever control measures.

## Abbreviations

DHS: Demographic and health surveys
MCMC: Markov Chain Monte Carlo
MDP: Millennium development project
GPS: Global positioning system
ZDHS: Zimbabwe demographic and health survey
ZNHSCP: Zimbabwe national household survey capability programme
EA: Enumeration area
CCA: Child’s current age
MABFC: Mother’s age at birth of first child
GAM: Geoadditive model
DIC: Deviance information criterion
WHO: World health organization
